# Artificial Intelligence for Detecting Pulmonary Embolisms *via* CT: A Workflow-oriented Implementation

**DOI:** 10.2174/0115734056367860250630072749

**Published:** 2025-07-23

**Authors:** Selim Abed, Klaus Hergan, Jan Dörrenberg, Lucas Brandstetter, Marcus Lauschmann

**Affiliations:** 1 Department of Radiology, University Hospital Salzburg, Paracelsus Medical University, 5020 Salzburg, Austria

**Keywords:** Lung, Pulmonary embolism, Deep learning and artificial intelligence, CTPAs, Patient care, Emergency radiology settings

## Abstract

**Introduction:**

Detecting Pulmonary Embolism (PE) is critical for effective patient care, and Artificial Intelligence (AI) has shown promise in supporting radiologists in this task. Integrating AI into radiology workflows requires not only evaluation of its diagnostic accuracy but also assessment of its acceptance among clinical staff.

**Objective:**

This study aims to evaluate the performance of an AI algorithm in detecting pulmonary embolisms (PEs) on contrast-enhanced computed tomography pulmonary angiograms (CTPAs) and to assess the level of acceptance of the algorithm among radiology department staff.

**Methods:**

This retrospective study analyzed anonymized computed tomography pulmonary angiography (CTPA) data from a university clinic. Surveys were conducted at three and nine months after the implementation of a commercially available AI algorithm designed to flag CTPA scans with suspected PE. A thoracic radiologist and a cardiac radiologist served as the reference standard for evaluating the performance of the algorithm. The AI analyzed 59 CTPA cases during the initial evaluation and 46 cases in the follow-up assessment.

**Results:**

In the first evaluation, the AI algorithm demonstrated a sensitivity of 84.6% and a specificity of 94.3%. By the second evaluation, its performance had improved, achieving a sensitivity of 90.9% and a specificity of 96.7%. Radiologists’ acceptance of the AI tool increased over time. Nevertheless, despite this growing acceptance, many radiologists expressed a preference for hiring an additional physician over adopting the AI solution if the costs were comparable.

**Discussion:**

Our study demonstrated high sensitivity and specificity of the AI algorithm, with improved performance over time and a reduced rate of unanalyzed scans. These improvements likely reflect both algorithmic refinement and better data integration. Departmental feedback indicated growing user confidence and trust in the tool. However, many radiologists continued to prefer the addition of a resident over reliance on the algorithm. Overall, the AI showed promise as a supportive “second-look” tool in emergency radiology settings.

**Conclusion:**

The AI algorithm demonstrated diagnostic performance comparable to that reported in similar studies for detecting PE on CTPA, with both sensitivity and specificity showing improvement over time. Radiologists’ acceptance of the algorithm increased throughout the study period, underscoring its potential as a complementary tool to physician expertise in clinical practice.

## 1 INTRODUCTION

The annual incidence of pulmonary embolism (PE) ranges from 39 to 115 cases per 100,000 population [1]. In the United States, PE accounts for up to 300,000 deaths annually [2]. Moreover, Martin *et al.* reported a rising incidence of PE in the United States between 1999 and 2018 [3]. In Europe, among 370,000 venous thromboembolism-related deaths across a population of 454 million, 34% of patients died suddenly or within hours of the acute event, before treatment could be initiated or become effective. Of the remaining cases, 59% were diagnosed with PE post-mortem, while only 7% of early deaths were correctly attributed to PE before the patient’s death [4]. However, PE-related mortality varies significantly depending on the severity of the condition [5].

Computed tomography pulmonary angiography (CTPA) is considered the gold standard for diagnosing PE due to its high sensitivity, resulting in a low false negative rate [6]. The PIOPED II trial, the largest study to date, reported a sensitivity of 83% and a specificity of 96% for CTPA [7, 8]. In contrast, Winer-Muram *et al.* found that CTPA has an accuracy of 91% in detecting PE [9]. CTPA also provides superior spatial resolution and allows for multi-planar reconstruction, enhancing diagnostic confidence [10]. Due to the diagnostic strength of CT and CTPA, their use has increased significantly in the United States in recent years [11].

Artificial intelligence (AI) in medical imaging is advancing rapidly, with notable improvements in accuracy and precision. Foundational models, such as U-Net, laid the groundwork for medical image segmentation, while newer architectures, like state-space models (SSMs) and Mamba-based networks, have introduced more advanced capabilities [12, 13]. Hybrid models, such as Mamba-UNet, have demonstrated enhanced segmentation accuracy, while adaptive frameworks, like S2VNet, improve the generalizability of AI across various tasks [14, 15]. As AI systems become increasingly sophisticated, their role is evolving from simple automation to intelligent collaboration with radiologists, supporting and enhancing clinical decision-making [16]. Emerging AI approaches that integrate deep learning and machine learning techniques have also shown high accuracy in detecting PE and prioritizing urgent cases [17].

Our radiology department implemented an FDA-approved and CE-marked deep learning algorithm designed to detect PE. The software alerts radiologists through a pop-up notification, enabling them to reprioritize their worklist based on the algorithm’s findings. Additionally, the algorithm highlights suspected PE locations using a heat map, assisting in the interpretation of the scan.

We hypothesized that the algorithm would demonstrate diagnostic performance comparable to that reported in previous studies and that radiologists would perceive benefits from the reprioritization of acute findings. To test this, we integrated the algorithm to run in the background for all CTPA interpretations. Additionally, we selected smaller random samples to evaluate the algorithm’s performance, using a board-certified thoracic radiologist as the reference standard. Two surveys were also conducted to assess radiologists’ acceptance of the algorithm.

## 2 MATERIALS AND METHODS

We obtained approval from the Institutional Review Board (IRB) for this retrospective study.

### 2.1 Study Design

Since August 2021, we have used a commercially available AI-based algorithm for the automatic detection of PE in CTPA scans. All patients who underwent CTPA at the University Hospital Salzburg between August 2021 and April 2022 were included, regardless of clinical indication or suspicion of PE. CTPA scans were excluded if they could not be processed by the AI algorithm due to suboptimal image quality, discrepancies in patient metadata (*e.g*., name or date inconsistencies), or technical incompatibility with the software. After applying these exclusion criteria, 46 CTPA scans were available for analysis during the first evaluation period (October 2021) and 40 scans during the second evaluation period (April 2022).

In October 2021, we conducted a retrospective analysis of CTPA scans from the preceding three weeks to evaluate the performance of the AI algorithm. Following this analysis, a survey was administered to assess radiologists’ acceptance of the algorithm. A second retrospective analysis and survey were performed six months later, in April 2022, using the same methodology. For both analyses, a subspecialized thoracic radiologist and a subspecialized cardiac radiologist jointly reviewed the CTPA scans to establish the ground truth. They reached a consensus on each case, having access to prior imaging, scan requests, and relevant laboratory values to support their interpretation. The radiologists were blinded to the AI algorithm’s results. False negatives were defined as cases where the algorithm failed to detect a PE identified by the radiologists, while false positives were cases where the algorithm indicated a PE that the radiologists did not identify. Discrepancies were instances in which the original radiology report did not indicate a PE, but the algorithm flagged the scan as positive.

### 2.2 AI Algorithm

**Fig. (1):**
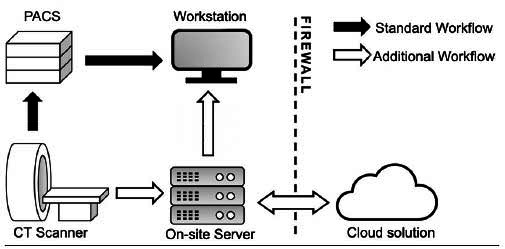
Diagram illustrating the architecture of the algorithm.

The AI algorithm used in this study (Aidoc Medical, Tel Aviv, Israel) is a deep learning model designed to assist in the detection of PE on CTPA scans. The algorithm is both FDA-approved and CE-marked. It was integrated into the institution’s Picture Archiving and Communication System (PACS) and Radiology Information System (RIS). Based on the ResNet architecture [18], this cloud-based algorithm automatically analyzes eligible imaging studies immediately upon acquisition. Before transmission to the provider for analysis, scans are pseudonymized to protect patient identity (Fig. **1**).

**Fig. (2):**
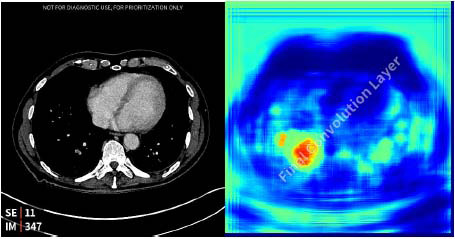
The algorithm correctly identifies and highlights a PE using computer vision.

Once the algorithm completes its analysis, the scans and corresponding results are returned, and the hospital’s IT department reverses the pseudonymization. The results are then integrated into the radiologist’s reading worklist within the RIS. Positive cases are highlighted in color on the worklist and accompanied by a pop-up notification. Additionally, a heatmap indicating the suspected abnormality is sent to the PACS to assist with interpretation (Fig. **2**).

### 2.3 Statistical Analysis

For the statistical analysis, we calculated sensitivity, specificity, Positive Predictive Value (PPV), and Negative Predictive Value (NPV). All statistical analyses were performed using SPSS.

## 3 RESULTS

### 3.1 First Retrospective Analysis

A total of 59 CTPA scans were reviewed during the first analysis period, from October 22, 2021 (22.09.2021) to October 14, 2021 (14.10.2021), for the detection of PE. Of these, 13 scans were not analyzed by the algorithm due to suboptimal image quality, discrepancies in patient information (*e.g*., name or date), or other technical issues. This corresponds to 22% of the total scans not being analyzed by the algorithm. The thoracic radiologist interpreted 13 out of 59 scans (22%) as positive for PE and 46 out of 59 as negative. Among the unanalyzed scans, 2 out of 59 (3.4%) were positive for PE, while 7 out of 59 (11.9%) were negative. The CTPA scans included PEs ranging from central to subsegmental locations. The results of the algorithm’s performance in the first analysis are presented in Table **1a**.

### 3.2 Second Retrospective Analysis

A total of 46 CTPA scans were reviewed during the second analysis period in April 2022 (04.04.2022 to 24.04.2022) for the detection of PE. Of these, 6 scans were not analyzed by the algorithm due to suboptimal image quality, discrepancies in patient information (*e.g*., name or date), or other technical issues. This represents 13% of the total scans, a lower rate of non-analysis compared with the first analysis. The thoracic radiologist interpreted 12 out of 46 scans (26%) as positive for PE and 34 out of 46 (74%) as negative. Among the unanalyzed scans, 1 (2.2%) was positive and 1 was negative for PE. The CTPA scans included PEs ranging from central to subsegmental. The results of the algorithm’s performance in the second analysis are presented in Table **1b**.

### 3.3 First Survey

Twenty-four out of 40 radiologists participated in the first survey. The remaining radiologists did not participate due to various reasons, including lack of exposure to the AI system, not having interpreted CTPAs, as well as vacation or sick leave. The survey was conducted six months after daily use of the algorithm began. The results of the first survey are summarized in Table **2a**.

### 3.4 Second Survey

After another six months, 9 out of 40 radiologists participated in the second survey. Not all radiologists were included due to various factors, including limited exposure to the AI system, not interpreting CTPA scans, vacation or sick leave, and staff turnover (radiologists leaving or new hires who were excluded from the survey). This survey reflects radiologists’ experiences following an additional six months of daily algorithm use. The results of the second survey are summarized in Table **2b**.

## 4 DISCUSSION

### 4.1 Sensitivity, Specificity, and Error Rate

In our first retrospective analysis, the algorithm demonstrated a sensitivity of 84.6% and a specificity of 94.3%. In the second analysis, sensitivity increased to 90.9% and specificity to 96.7%. These values were calculated excluding scans that were not analyzed by the algorithm. Under these conditions, the algorithm’s diagnostic performance was comparable to that reported in similar studies. We observed an improvement in the algorithm’s performance throughout the study, suggesting potential adaptation based on feedback.

Additionally, the number of CTPA scans not processed by the algorithm decreased from 13 out of 59 in the first period to 6 out of 46 in the second, indicating improved compatibility or system stability over time. This represents a decrease in non-analyzed or non-compatible studies from 22% to 13%. While this may suggest an improvement in the algorithm’s ability to process technically suboptimal scans, some exclusions were due to discrepancies in patient metadata (*e.g*., name or date inconsistencies) or other administrative errors. These factors are unrelated to the algorithm itself and instead reflect limitations within the IT infrastructure and data management processes. As a result, there is an inherent limitation in attributing the reduction in non-analyzed scans solely to improvements in the algorithm’s adaptability. Both algorithmic advancements and improved clinical data management likely contributed to the observed reduction in non-analyzed scans. However, since the specific reasons for exclusion were not systematically documented, it is not possible to precisely quantify the relative impact of each factor. Assuming both played a role, these findings suggest that the algorithm is capable of adapting to a new clinical environment and becoming more attuned to the specific characteristics of scans within our institution.

**Fig. (3):**
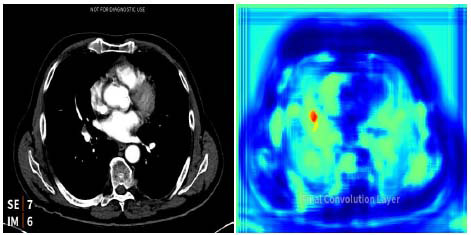
Lymphatic tissue - incorrectly labeled as a PE (false positive).

Excluding non-compatible scans and focusing only on those not analyzed by the algorithm, the false negative rate (*i.e*., not analyzed but positive for PE) decreased from 2 out of 59 (3.4%) in the first period to 1 out of 46 (2.2%) in the second. Similarly, the rate of scans not analyzed or negative for PE decreased from 7 out of 59 (11.9%) to 1 out of 46 (2.2%). Previous studies evaluating similar algorithms have reported sensitivities ranging from 86.6% to 100% and specificities between 89% and 95.5% [19-23]. Compared with these findings, our calculated sensitivity was slightly lower in the first period, while the other performance metrics were consistent with those reported in the literature for the same algorithm. We observed that the AI algorithm occasionally generated false positives, most commonly when lymphatic tissue or vascular bifurcations were misinterpreted as emboli (Fig. **3**). False negatives were more frequently associated with scans exhibiting suboptimal contrast timing or with small, subsegmental emboli that are more difficult to detect.

### 4.2 Time and Place

It has been shown that 95% of PE in the United States is managed in hospital settings [24]. Consequently, most PEs are diagnosed using CTPA, highlighting the critical need for accurate and timely reporting. Kennedy *et al.* found that emergency medicine physicians considered the optimal time for preliminary CTPA interpretation to be 20 minutes, while radiology department chairs deemed 60 minutes acceptable [25]. Prompt initiation of anticoagulant therapy is essential for the effective treatment of acute PE [26], and delayed administration has been causally linked to fatal outcomes [27]. This highlights not only the importance of diagnostic accuracy but also the critical role of reporting speed in influencing patient outcomes in the management of PE. Long-term mortality rates for patients with PE vary significantly depending on the data source. The three-month mortality rate after anticoagulant treatment is reported to be below 5% in randomized clinical trials, between 5% and 10% in diagnostic outcome studies, and approximately 15% in most registries and cohort studies [28].

### 4.3 Possible Benefits and Risks of an Algorithm

There are multiple applications and benefits to implementing such an AI program. It enhances workflow by reprioritizing scans with acute findings, such as PE, which is particularly valuable in emergency settings. AI algorithms have been shown to effectively reorganize radiologists’ reading worklists to prioritize critical cases [29]. Given the significant increase in CT scan requests from emergency departments in recent years [30], the use of AI can improve efficiency by enabling rapid screening of large image volumes, thereby reducing the radiologists’ workload. Furthermore, the demand for rapid turnaround times in emergency settings increases the risk of interpretive errors an area where AI support can help mitigate mistakes. Our hospital has implemented a PE Response Team (PERT), making rapid and accurate diagnosis essential. This further underscores the value of an AI algorithm used as a “second look” tool. AI algorithms in this role have been shown to improve the accuracy of reporting by increasing the rate of correct diagnoses [31-33]. Our radiology department is currently unable to provide a board-certified radiologist to review every CTPA interpreted by a resident. Several studies have demonstrated that attending radiologists have higher detection rates compared with residents [34-38], highlighting the potential of such AI programs to provide valuable support, particularly for less experienced radiologists. The algorithm can also serve as a feedback and teaching tool, especially when it identifies a PE missed by the interpreting radiologist. This is particularly relevant for incidental PEs detected through natural language processing (NLP). In some cases, the algorithm may even help reveal potential diagnostic blind spots, which could be incorporated into standardized review protocols to enhance patient care.

There are also several risks associated with the use of such an algorithm. As demonstrated in our study, the algorithm exhibits notable false-positive and false-negative rates. One key limitation is its lack of interpretability when the interpreting radiologist disagrees with the algorithm’s result, the AI cannot explain its decision, making certain findings difficult to assess. In our study, we observed that the algorithm missed PEs on suboptimal CTPA scans, which contrasts with the findings of Ebrahimian *et al.* [21]. We also recognize the risk of over-reliance on the algorithm, especially given that it may not be capable of analyzing all scans, particularly those with suboptimal image quality. Furthermore, AI algorithms primarily rely on pattern recognition and are unable to incorporate clinical context, which can contribute to diagnostic inaccuracies.

### 4.4 Opinion of the Department

As noted above, we conducted two surveys six months apart to assess radiologists’ acceptance of the algorithm. In the first survey, 45.87% of respondents agreed or strongly agreed that the algorithm made them feel more secure in their reporting. This increased to 77.8% in the second survey, suggesting that radiologists became more comfortable relying on the algorithm over time. Additionally, only 4.2% agreed or strongly agreed in the first survey that the algorithm made them feel pressed for time this dropped to 0% in the second survey, indicating that the algorithm was not perceived as an added source of stress. In the first survey, 62.47% of respondents agreed or strongly agreed that the algorithm should continue to be used as a tool within the department. This figure declined slightly to 55.5% in the second survey. Meanwhile, the proportion of radiologists who agreed or strongly agreed that hiring an additional resident would be more beneficial than using the algorithm increased from 70.8% to 88.9%. Given that the cost of employing a resident is equivalent to that of implementing the algorithm, this is a noteworthy finding that highlights radiologists’ preference for human expertise when resources are limited.

In the first survey, 16.7% of respondents felt that the algorithm correctly identified positive cases only 25–50% of the time. In the second survey, this figure dropped to 0%, with all radiologists indicating that the algorithm correctly reported positive findings in at least 50% of cases. This shift may indicate that, over time, radiologists developed greater trust in the algorithm's capabilities and gained a better understanding of its strengths and limitations through continued use.

In summary, over time, radiologists became more accustomed to and comfortable with the algorithm, increasingly integrating it into their daily workflow. However, despite growing familiarity and trust, they consistently preferred the addition of a resident over the use of the algorithm, particularly when considering the comparable cost.

### 4.5 Strengths and Weaknesses of the Study

The surveys conducted in our department at two different time points demonstrate a generally positive outlook toward AI adoption among radiologists, with a clear trend of increasing acceptance over time. We calculated sensitivity and specificity to compare our results with those reported in existing studies and found comparable performance. To resolve discrepancies between the algorithm’s interpretations and the radiologists’ assessments, a subspecialized thoracic radiologist and a subspecialized cardiac radiologist were consulted for consensus.

Several limitations must be considered when interpreting our results. First, the study was conducted at a single hospital, which limits the generalizability of our findings to other institutions or clinical settings. Second, although our sensitivity and specificity values align with those reported in other studies, the sample size in our analysis is relatively small. Another notable limitation is that several CTPA scans were not analyzed by the algorithm, making it difficult to determine how to classify these cases in the statistical evaluation. A key consideration is whether unanalyzed cases should be automatically counted as false results, regardless of the presence or absence of PE. In this regard, our findings differ from those of Ebrahimian et al., who reported that “suboptimal CTPA examinations do not impair the performance of [the] PE-AI triage model ” [21]. In our study, suboptimal scans were more likely to be excluded by the algorithm, and several of these did contain PE, suggesting a potential limitation in its robustness. Additionally, we did not assess follow-up imaging for patients with missed PEs, as this fell outside the scope of the study. Therefore, we cannot determine whether the missed PEs were subsequently detected in later scans.

The number of radiologists who participated in the surveys was relatively low and further declined in the second round. This may be attributed to a variety of factors, including general skepticism toward AI, absence due to vacation or sick leave, staff turnover (radiologists leaving or new ones joining who were not present for both surveys), and exclusion of those who did not interpret CTPA scans.

## CONCLUSION

Our study found that the algorithm’s sensitivity and specificity were comparable to those reported in similar studies. Consistent with prior research, we observed an improvement in both metrics over time, suggesting ongoing adaptation. The algorithm was able to detect small PEs that may have been overlooked by interpreting radiologists, though these were often located in very distal pulmonary arteries and may not have been clinically significant. Additionally, the algorithm demonstrated improved compatibility with imaging data throughout the study period, as evidenced by a reduced rate of unanalyzed or uninterpreted CTPA scans, further supporting its adaptability to a new clinical environment.

The algorithm serves as a valuable adjunct tool, particularly in high-volume radiology settings, by reprioritizing scans and potentially reducing reporting delays. This may facilitate earlier initiation of anticoagulation therapy, thereby improving patient outcomes. Our findings indicate growing acceptance of the algorithm over time; however, an increasing number of radiologists expressed a preference for hiring an additional resident, especially given the comparable annual cost. AI should be viewed as a complement to radiologists, enhancing workflow and supporting clinical decision-making, rather than replacing human expertise. To maximize its utility, careful integration of AI algorithms and close collaboration with the IT department of the hospital are essential.

## AUTHORS’ CONTRIBUTIONS

The authors confirm their contributions to the paper as follows: Writing, review, and formal analysis: SA; conceptualization and supervision: KH; editing and review: JD; methodology and editing: LB; validation and data curation: ML. All authors reviewed the results and approved the final version of the manuscript for submission.

## Figures and Tables

**Table 1a:** Statistical analysis of the CTPAs analyzed during the first period in October 2021 (22.09.2021 to 14.10.2021), using the board-certified thoracic radiologist as the ground truth. This analysis excludes CTPAs that were not analyzed and were negative for PE, as well as those deemed non-compatible by the algorithm.

n = 48	True Positive	True Negative	False Positive	False Negative	Sensitivity	Specificity	Positive Predictive Value	Negative Predictive Value
The algorithm’s performance	11 (22,9%)	33 (68,8%)	2 (4,2%)	2 (4,2%)	85%	94%	85%	94,28%

**Table 1b:** Statistical analysis of the CTPAs analyzed during the second period in April 2022, using the board-certified thoracic radiologist as the gold standard. This analysis excludes CTPAs that were not analyzed and were negative for PE, as well as those deemed non-compatible by the algorithm.

**n = 41**	**True Positive**	**True Negative**	**False Positive**	**False Negative**	**Sensitivity**	**Specificity**	**Positive Predictive Value**	**Negative Predictive Value**
The algorithm’s performance	10	29	1	1	91%	97%	91%	97%

**Table 2a:** Results of the first survey, conducted in October 2021 after three months of using the AI algorithm, showing responses based on levels of agreement.

n = 27	**Strongly Agree**	**Agree**	**Somewhat Agree**	**Disagree**	**Strongly Disagree**
**The algorithm makes me feel more secure in my reporting**	4 (16,7%)	7 (29,17%)	7 (29,2%)	4 (16,7%)	2 (8,3%)
**The algorithm is practicable for use with the PACS**	3 (12,5%)	9 (37,5%)	8 (33,3%)	4 (16,7%)	0 (0%)
**The algorithm is increasing the speed of my reporting**	2 (8,3%)	2 (8,3%)	4 (16,7%)	11 (45,8%)	5 (20,8%)
**The algorithm makes me feel pressed for time**	0 (0%)	1 (4,2%)	2 (8,3%)	5 (20,8%)	16 (66,7%)
**The algorithm detected a PE that I overlooked**	4 (16,7%)	2 (8,3%)	2 (8,3%)	9 (37,5%)	7 (29,17%)
**Should the algorithm continue to be used as a tool in the department?**	7 (29,17%)	8 (33,3%)	4 (16,7%)	3 (12,5%)	2 (8,3%)
**Considering the added value of the algorithm. Would an additional resident position be more useful?**	14 (58,3%)	3 (12,5%)	3 (12,5%)	2 (8,3%)	2 (8,3%)

**Table 2b:** Results of the second survey, conducted in May 2022 after nine months of using the AI algorithm, showing responses based on levels of agreement.

n = 9	**Strongly Agree**	**Agree**	**Somewhat Agree**	**Disagree**	**Strongly Disagree**
**The algorithm makes me feel more secure in my reporting**	2 (22,2%)	5 (55,6%)	1 (11,1%)	0	1 (11,1%)
**The algorithm is practicable for use with the IMPAX**	3 (33,3%)	3 (33,3%)	2 (22,2%)	1 (11,1%)	0
**The algorithm is increasing the speed of my reporting**	1 (11,1%)	3 (33,3%)	0	0	5 (55,6%)
**The algorithm makes me feel pressed for time**	0	0	1 (11,1%)	4 (44,4%)	4 (44,4%)
**The algorithm detected a PE that I overlooked**	1 (11,1%)	0	1 (11,1%)	4 (44,4%)	3 (33,3%)
**Should the algorithm continue to be used as a tool in the department?**	3 (33,3%)	2 (22,2%)	2 (22,2%)	1 (11,1%)	1 (11,1%)
**Considering the added value of the algorithm. Would an additional resident position be more useful?**	7 (77,8%)	1 (11,1%)	0	0	1 (11,1%)

## Data Availability

All data generated or analyzed during this study are included in this published article.

## LIST OF ABBREVIATIONS

AI: Artificial Intelligence
PE: Pulmonary Embolism
CTPA: CT Pulmonary Angiography
IRB: Institutional Review Board

## STANDARDS OF REPORTING

STROBE guidelines were followed.
